# Malaria and Scrofula as Respective Causes of Acute and Chronic Skin Diseases

**Published:** 1882-12

**Authors:** 


### MALARIA AND SCROFULA AS RESPECTIVE CAUSES OF ACUTE AND CHRONIC SKIN DISEASES.

We have been favored by Dr. Lunsford P. Yandell, one of the editors of the *Louisville Medical News*, with an advance sheet of the *News* of November 25th, wherein he seeks to set himself right with the profession in regard to the position he has occupied for several years concerning the causative relation which exists, according to his theory, between malaria and acute skin diseases on the one hand, and scrofula and chronic skin diseases on the other; and the coexistence of these two prime etiological factors in combination as the basis of some of the more obstinate forms of the dermatoses.

This special explanation is thought to be necessary on account of a paragraph which has recently been going the rounds of the medical press to the effect that Dr. Yandell "attributes all skin diseases to malaria. Quinine is a specific for malaria; ergo, quinine is the remedy for all skin eruptions."

We have observed, for several weeks past, the item above quoted, in the columns of a number of our exchanges, but we have not given it place in the REGISTER because we felt that the squib was scarcely just to the gentleman whose opinion it professed to represent. Nevertheless, we cheerfully contribute our assistance in vindication of our esteemed confrere, not only as a matter of personal interest, but, as he says, "of scientific interest to the profession."

Dr. Yandell's position, as defined by himself, is simply this: "Malaria is the chief source of acute skin disease. Scrofula is the chief source of chronic skin disease. The more inveterate cases of skin disease are often due to the coexistence of these two things. The specific exanthems, of course, are not included here, but I contend that their progress and termination are often largely influenced by the presence of malaria, or struma. I do not claim that malaria and struma are the soul causes of the dermatoses. Indeed, many of the dermatoses may exist independently of malaria or struma, and most frequently some exciting cause is necessary to develop the cutaneous eruption. Among the exciting causes are irritants, injuries, insufficient, or improper ingesta, vicissitudes of temperature, alcohol, dentition, menstruation, parturition, lactation, etc.," and he maintains that the success of antimalarial and antistrumous remedies, in this class of diseases, is confirmatory of his views.

We have referred to this subject merely for the purpose indicated above, but, before dismissing it, two appropriate observations occur to us, namely: First, that malaria is a comprehensive, elastic and indefinite term, and that it literally means a great deal and includes a vast variety of bad air. Second, that all diseases occurring in malarial districts—the term is used here in its restricted and commonly accepted sense—are liable to be impressed by that peculiar poison, and consequently to manifest periodicity or other evidences of malarial complication.

It has appeared to us that many observers have overlooked or under-estimated this important fact in their published writings, especially as relates to therapeutics—that is to say, the antimalarial treatment which would be essential in one locality would be uncalled for or improper in another, not subjected to like influences.

